# Parathyroid Hormone-Related Peptide (1-36) Enhances Beta Cell Regeneration and Increases Beta Cell Mass in a Mouse Model of Partial Pancreatectomy

**DOI:** 10.1371/journal.pone.0158414

**Published:** 2016-07-08

**Authors:** Anaïs Mozar, Hugo Lin, Katoura Williams, Connie Chin, Rosemary Li, Nagesha Guthalu Kondegowda, Andrew F. Stewart, Adolfo Garcia-Ocaña, Rupangi Chhaya Vasavada

**Affiliations:** 1 Diabetes, Obesity and Metabolism Institute, Icahn School of Medicine at Mount Sinai, New York, New York, United States of America; 2 Division of Endocrinology, University of Pittsburgh, Pittsburgh, Pennsylvania, United States of America; CHA University, REPUBLIC OF KOREA

## Abstract

**Aims/Hypothesis:**

Finding ways to stimulate the regeneration of endogenous pancreatic beta cells is an important goal in the treatment of diabetes. Parathyroid hormone-related protein (PTHrP), the full-length (1–139) and amino-terminal (1–36) peptides, enhance beta cell function, proliferation, and survival. Therefore, we hypothesize that PTHrP(1–36) has the potential to regenerate endogenous beta cells.

**Methods:**

The partial pancreatectomy (PPx) mouse model of beta cell injury was used to test this hypothesis. Male Balb/c mice underwent either sham-operation or PPx, and were subsequently injected with PTHrP(1–36) (160μg/kg) or vehicle (veh), for 7, 30, or 90 days. The four groups of mice, sham-veh, sham-PTHrP, PPx-veh, and PPx-PTHrP were assessed for PTHrP and receptor expression, and glucose and beta cell homeostasis.

**Results:**

PTHrP-receptor, but not the ligand, was significantly up-regulated in islets from mice that underwent PPx compared to sham-operated mice. This suggests that exogenous PTHrP could further enhance beta cell regeneration after PPx. PTHrP did not significantly affect body weight, blood glucose, plasma insulin, or insulin sensitivity, in either sham or PPx mice. Glucose tolerance improved in the PPx-PTHrP versus PPx-veh mice only in the early stages of treatment. As hypothesized, there was a significant increase in beta cell proliferation in PPx-PTHrP mice at days 7 and 30; however, this was normalized by day 90, compared to PPx-veh mice. Enhanced beta cell proliferation translated to a marked increase in beta cell mass at day 90, in PPx-PTHrP versus PPx-veh mice.

**Conclusions:**

PTHrP(1–36) significantly enhances beta cell regeneration through increased beta cell proliferation and beta cell mass after PPx. Future studies will determine the potential of PTHrP to enhance functional beta cell mass in the setting of diabetes.

## Introduction

A reduction in functional pancreatic beta cell mass contributes to all forms of diabetes. One approach to correct the functional beta cell mass deficit in diabetes is through pancreas or islet transplantation. However, this strategy has limited applicability due to shortage of organ donors, stress-related damage to transplanted tissue, and the negative impact of the immunosuppressive regimen [1–3]. Regeneration of a patient’s own beta cells, either through neogenesis, or proliferation of pre-existing beta cells, and/or preventing the further loss of beta cells, are promising alternative approaches to replenish the diminishing beta cell mass in diabetic patients. Although the rate of basal beta cell proliferation is low [4–6], there is evidence that beta cell replication can be induced in response to metabolic demand, such as pregnancy, obesity, aging, or partial pancreatectomy (PPx) in rodents [7–9]. This suggests that external stimuli could be used to further induce endogenous beta cell replication, and enhance beta cell mass.

Parathyroid hormone-related protein (PTHrP) and its seven transmembrane G-protein coupled PTH-1 receptor (PTH1R) are expressed in every tissue in the body, including the pancreatic beta cell [10–13]. PTHrP expression is increased in insulinomas [13–15], suggesting that PTHrP could induce beta cell proliferation. Indeed, transgenic mice overexpressing full-length PTHrP(1–139) in the beta cell, under the rat insulin promoter (RIP), display hyperinsulinemia, hypoglycemia, beta cell hyperplasia due to increased proliferation, with a resultant increase in beta cell mass. RIP-*PTHrP* transgenic mice are resistant to streptozotocin-induced diabetes and beta cell death [12, 16–19].

Full-length PTHrP(1–139) is post-translationally processed to form amino-terminal (1–36), mid-region (38–94), and carboxyl-terminal (107–139) peptides, each having specific functions in other cell types [10–11, 20]. Only amino-terminal containing PTHrP peptides bind and activate the PTH1R [10–11]. Of the various PTHrP peptides, amino-terminal peptide PTHrP(1–36), added exogenously *in vitro*, is sufficient to increase proliferation [19, 21–22], and improve survival against streptozotocin and nutrient-deprivation induced cell death, in cell-lines and primary rodent beta cells [18]. Furthermore, PTHrP(1–36) also enhances beta cell function, increasing insulin mRNA and protein, as well as glucose-stimulated insulin secretion (GSIS) in rodent beta cells *in vitro* [21–23]. Based on these *in vitro* findings, in a previous study we tested whether PTHrP(1–36) would have similar beneficial effects on the beta cell *in vivo*. Indeed, in normal Balb/c mice PTHrP(1–36) stimulated beta cell proliferation and augmented beta cell mass, without negatively affecting beta cell function or survival, when administered systemically under basal conditions [24].

The present study examines the *regenerative potential* of PTHrP *in vivo*. In other words, can PTHrP(1–36) stimulate beta cell proliferation and regeneration in a model of beta cell deficiency? We used the partial pancreatectomy (PPx) mouse model of beta cell injury to evaluate the effect of systemic administration of PTHrP(1–36) peptide for 7, 30, and 90 days. Glucose and beta cell homeostasis, as well as expression of PTHrP and PTH1R were assessed in PPx and sham-operated mice. PTHrP(1–36) substantially augmented the natural beta cell regeneration in the PPx mouse model, accelerating beta cell proliferation and increase in beta cell mass. These studies highlight the regenerative potential of PTHrP(1–36) for the beta cell.

## Materials and Methods

### PPx experimental design

**Fig 1:**
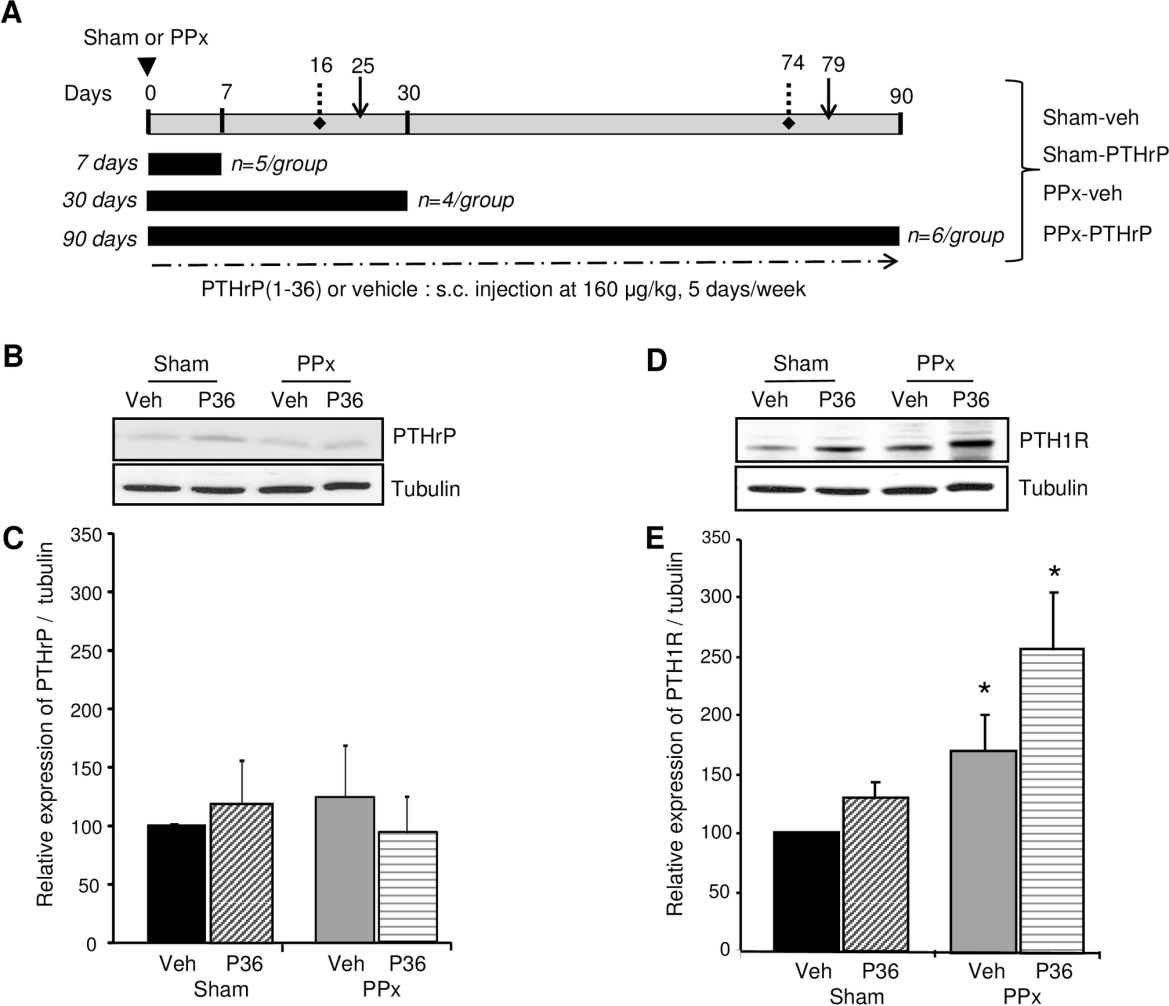
PTH1 receptor levels increase in islets of PPx mice. **(A)** PPx experimental design for analysis of glucose and beta cell homeostasis. Eight week-old male Balb/c mice underwent PPx or sham-operation at day 0, and were injected s.c. for 5 days/week with vehicle (veh) or PTHrP(1–36) (P36) at 160μg/kg body weight, resulting in four groups of mice: sham-veh, sham-PTHrP, PPx-veh, and PPx-PTHrP. The four groups of mice were treated for 7 days (n = 5 mice/group), 30 days (n = 4 mice/group), or 90 days (n = 6 mice/group). IPGTT was performed at days 25 and 79 (solid arrows) and ITT at days 16 and 74 (dotted arrows) on the four groups of mice. Representative western blot analysis of **(B)** PTHrP, and **(D)** PTH1R, with tubulin as loading control, in islets isolated from mice treated for 7 days. Western blot analysis was performed on a separate additional group of mice from those shown in Fig 1A. Quantification of the expression of **(C)** PTHrP/tubulin, and **(E)** PTH1R/tubulin ratios in the four groups of mice: sham-veh (black bars), sham-PTHrP (stippled bars), PPx-veh (grey bars) and PPx-PTHrP (horizontally-stippled bars) (n = 9–12 mice/group). Significance of * p<0.02 by ANOVA versus corresponding sham controls.

This study was carried out in strict accordance with the recommendations in the Guide for the Care and Use of Laboratory Animals of the National Institutes of Health. The protocol for this study was approved by the Committee on the Ethics of Animal Experiments of the University of Pittsburgh (Permit Number: 1101117A-1) and the Icahn School of Medicine at Mount Sinai (Permit Number: LA12-00304). All surgery was performed under ketamine and xylazine used for anesthesia, buprenorphine to alleviate pain, and all efforts were made to minimize suffering. Animals were monitored continuously after surgery until they were able to maintain sternal recumbency as required by the IACUC policy on surgical guidelines. Subsequently they were monitored twice a day for 3–5 days to administer analgesic and to ensure general health. The specific criteria used to monitor animal health included hunched posture, piloerection, abnormal feeding, drinking and ambulation. We did not have any mouse that was sick or that died during the experiment. If there were adverse signs that persisted 24h post-surgery, or a pronounced decrease in body weight (>20%), the mouse would be euthanized using carbon dioxide inhalation followed by cervical dislocation. 7–8 week old male Balb/c mice (Charles River, Wilmington, MA) were sham-operated or underwent PPx, and were given subcutaneous (s.c.) injections of either vehicle (veh) (10mM acetic acid) or PTHrP(1–36) peptide. Thus, mice were divided into four treatment groups: sham-veh, PPx-veh, sham-PTHrP or PPx-PTHrP. For PPx, portion of the pancreas (~40%) bordered by the spleen and the stomach was excised through an upper midline incision, keeping the mesenteric pancreas intact [25–26]. The rationale for the 40% PPx was to generate a model of reduced beta cell mass in the context of normal blood glucose. The sham-operated animals were handled similarly, but the pancreas was not removed. The mice were weighed and injected daily for 5 days/week, starting on the day of surgery (Day 0) up to 7, 30 or 90 days, receiving 90μl of phosphate buffered saline with either 10μl of vehicle, or 10μl of PTHrP(1–36) peptide at 160μg/kg body weight (Fig 1A). PTHrP(1–36) peptide was prepared using solid phase synthesis, purity documented by analytical reverse-phase HPLC, peptide structure and content confirmed using MALDI-TOF mass spectrometry and amino acid analysis, and assayed for activity using the SaOS-2 adenylyl cyclase assay as described previously [27]. The mice were given intraperitoneal (i.p.) injection of bromodeoxyuridine (BrdU) (Amersham Pharmacia Biotech, Piscataway, NJ) 50μg/g body weight, 6h before their pancreata were harvested. Islets were isolated for western blot analysis from mice treated for seven days.

### Glucose homeostasis

Blood glucose was measured twice a week on tail snips using a portable glucometer (Medisense, Bedford, MA). Intraperitoneal glucose tolerance test (IPGTT) was performed on days 25 and 79 in mice fasted for 16-18h injected with 2g glucose/kg body weight. Insulin tolerance test (ITT) was performed on days 16 and 74 in mice injected i.p. with 1.5U/kg of insulin (Lilly, Indianapolis, IN). Plasma insulin was measured on blood drawn on days 7, 30, and 90, using an insulin RIA (Linco, St Charles, MO).

### Pancreatic histomorphometry, islet size distribution, exocrine and beta cell proliferation

Pancreata were weighed, fixed in Bouin’s (Sigma, St Louis, MO), and paraffin embedded. Sections were stained for insulin using guinea pig anti-insulin antibody (Invitrogen, Carlsbad, CA) at 1:1000 dilution overnight at 4°C, and visualized using the link-label detection system and diaminobenzidine tetrahydrochloride substrate (BioGenex, San Ramon, CA). Histomorphometry was performed in a blinded way on at least 3–4 insulin-stained pancreatic sections per animal separated by 50μm each, using the Optimas software package. Beta cell mass was quantified as the ratio of the insulin-positive to total pancreatic area, multiplied by the pancreas weight. Islet size distribution including number of insulin-positive singlets and doublets was analyzed manually using an intraocular grid under a 10X objective to measure islet diameter. Exocrine cell proliferation was assessed on pancreatic sections stained for phosphohistone H3 (pHH3, 1:200, Millipore, Billerica, MA) analyzing an average of 3921±133 cells/mouse pancreas. Beta cell proliferation was quantified as percentage of BrdU-insulin- to total insulin-positive cells on pancreatic sections stained with antibodies against insulin (Dako, Carpinteria, CA) and BrdU (1:5 dilution) (Amersham Pharmacia Biotech), using an immunofluorescence secondary antibody, after antigen retrieval at 37°C for 30 min in 2N hydrochloric acid [19, 24].

### Islet isolation and western blot analysis

Islets were isolated from 7-day treated mice, plated in RPMI medium with 5.5mM glucose and 1% fetal bovine serum, immediately handpicked using a microscope grid, rinsed and frozen. Islet extracts (20μg) were analyzed by immunoblot using antibodies to PTH1R receptor (Covance, Richmond, California), PTHrP (EMD Millipore-Calbiochem, Billerica, MA), and tubulin (EMD Millipore-Calbiochem). Quantitative densitometry of digitalized blots was performed using the Image J program (National Institute of Health) [24, 25].

### Statistical analysis

Data are expressed as means ± standard error. To determine statistical significance one-way Analysis of variance (ANOVA) with post hoc Tukey Honestly Significant Difference (HSD) test (http://statistica.mooo.com) was used for comparison between more than two groups. Differences were considered significant at p≤0.05.

## Results

### PTH1 receptor is up-regulated in islets from PPx mice

To examine if PTHrP(1–36) could enhance beta cell regeneration after injury, we used PPx, a well characterized model of partial beta cell depletion that induces beta cell proliferation and regeneration under normoglycemic conditions [25–26]. Male Balb/c mice underwent sham-operation or PPx at day 0, and were injected s.c. with either vehicle or PTHrP(1–36) at 160μg/kg body weight for 5 days/week for 7, 30, or 90 days, resulting in four groups of mice: sham-veh, sham-PTHrP, PPx-veh, or PPx-PTHrP (Fig 1A). Body weight and blood glucose were measured twice a week; plasma insulin at the end of each treatment; and IPGTT and ITT were measured at the times indicated in Fig 1A.

We first examined whether endogenous expression of PTHrP or PTH1R is modulated by PPx, a model of beta cell regeneration. PTHrP ligand and its receptor, PTH1R, were analyzed by western blot analysis in the islets of sham and PPx mice treated with vehicle or PTHrP(1–36) at day 7 (Fig 1B–1E). There was no significant difference in the expression of PTHrP peptide among the four groups, sham-veh, PPx-veh, sham-PTHrP, and PPx-PTHrP mice (Fig 1B and 1C). PTHrP(1–36) treatment did not affect the levels of the endogenous receptor PTH1R in islets of sham or PPx mice when compared to their respective vehicle controls (Fig 1D and 1E), although there was a trend towards an increase in PPx-PTHrP versus PPx-Veh mice that was not significant. However, PPx caused a significant 70% increase in the abundance of PTH1R, in islets of PPx-veh versus sham-veh mice (Fig 1D and 1E). Similarly, there was a significant two-fold increase in the abundance of PTH1R protein in the PPx-PTHrP versus sham-PTHrP mice (Fig 1D and 1E). Together, this indicates that PPx *per se* causes an increase in PTH1R levels.

### Effect of PTHrP(1–36) on glucose homeostasis after PPx

**Fig 2:**
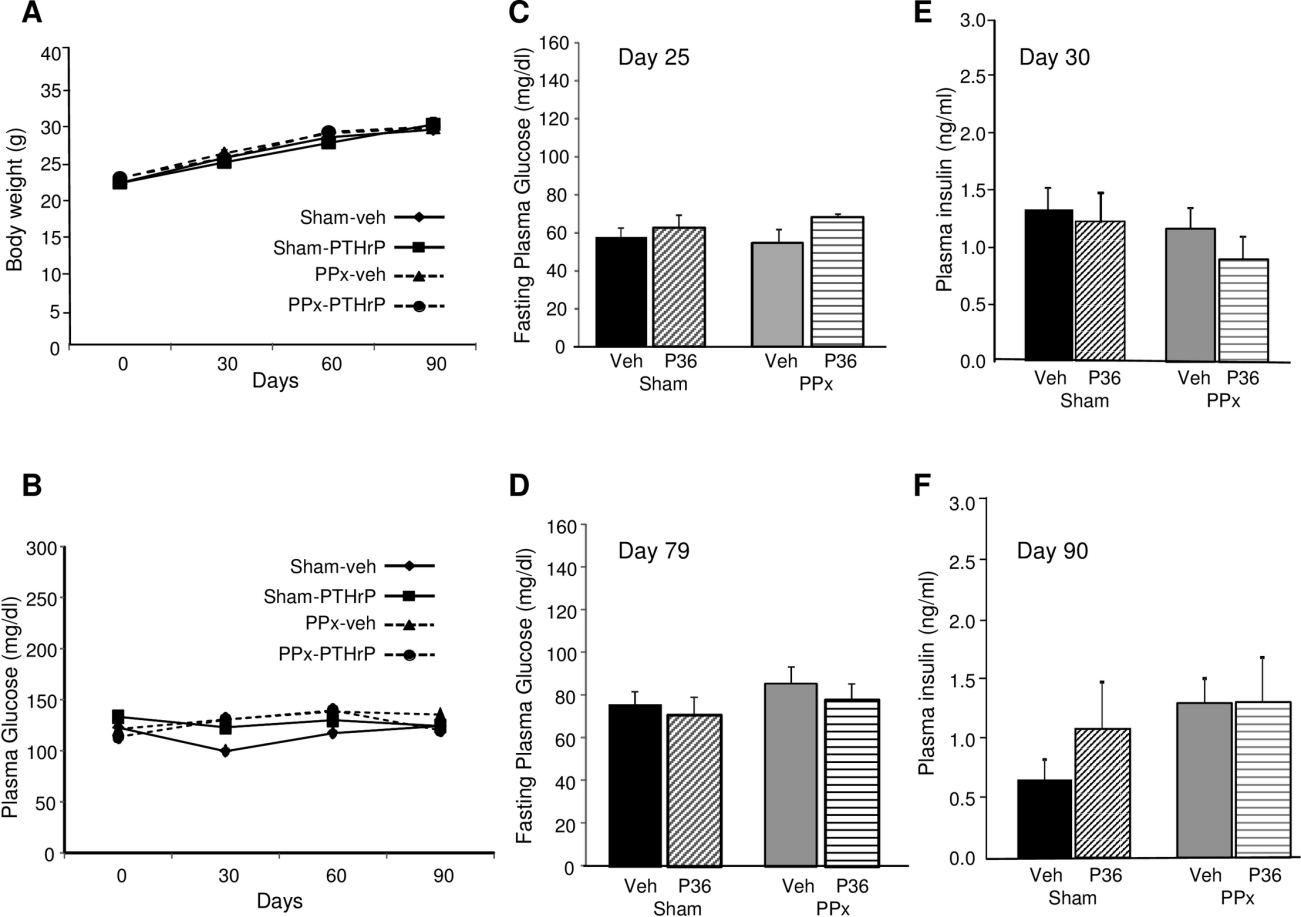
Body weight and glucose homeostasis. Average **(A)** body weight and **(B)** blood glucose values at 30-day intervals in the sham (solid line) -veh (diamond), -PTHrP (square), and PPx (dotted line) -veh (triangle), -PTHrP (circle) mice, treated for 90 days (n = 6 mice/group). Fasting plasma glucose at **(C)** day 25 and **(D)** day 79, plasma insulin at **(E)** day 30 and **(F)** day 90 in the four groups of mice treated for 30 days (C, E, n = 4 mice/group) or 90 days (D, F, n = 5 mice/group), as represented in Fig 1.

As expected [25–26], 40% PPx was associated with normal body weight (Fig 2A) and normal random non-fasting blood glucose (Fig 2B) in the PPx mice and the PTHrP-treated mice during the entire 90-day treatment period. The body weight and blood glucose levels did not differ amongst the four groups of mice treated for 7 and 30 days either (data not shown). Also, fasting blood glucose measured at day 25 in the 30 day treatment group (Fig 2C) and day 79 in the 90 day treatment group (Fig 2D) was not different among the four groups of mice. Together, this confirms that PPx did not induce hyperglycemia, and shows that PTHrP(1–36) treatment did not affect blood glucose in sham or PPx mice. There was no significant difference in the plasma insulin of the four groups of mice either at day 30 (Fig 2E) or at day 90 (Fig 2F).

**Fig 3:**
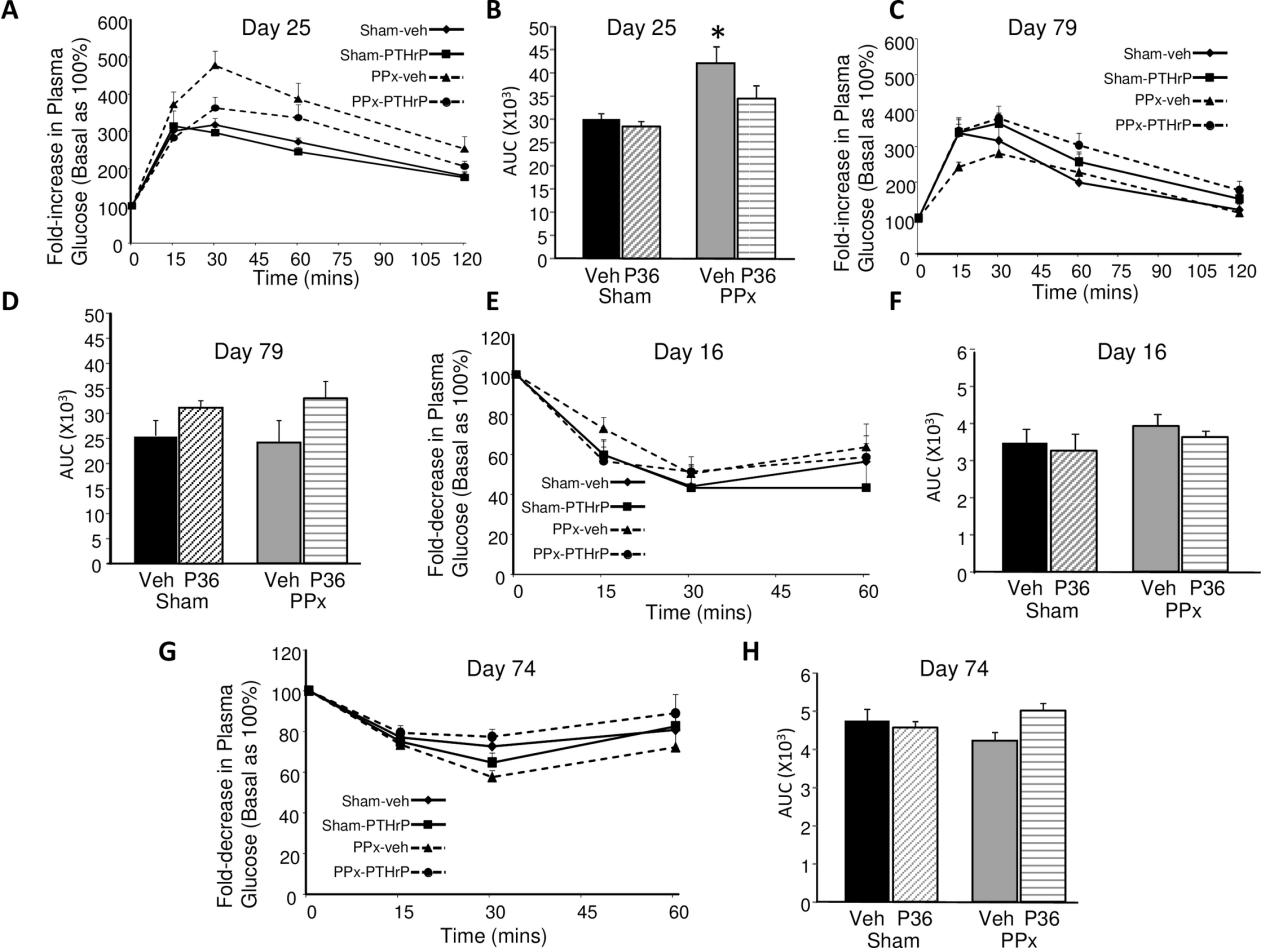
IPGTT and ITT. **(A,C)** IPGTT, **(B,D)** and area under the curve (AUC) for IPGTT performed on **(A,B)** day 25 and **(C,D)** day 79. **(E,G)** ITT, **(F,H)** and AUCs for ITT performed on **(E,F)** day16 and **(G,H)** day 74, in the four groups of mice, as represented in Fig 2 for the line graphs, and in Fig 1 for the bar graphs. The day 16 and day 25 procedures were performed on the 30 day treatment groups (n = 4 mice/group), and the day 74 and day 79 procedures were performed on the 90 day treatment groups (n = 6 mice/group). Y-axis depicts fold-change in plasma glucose relative to basal (time 0) taken as 100%. There was no significant difference in the basal plasma glucose among the four groups in all experiments. Significance of * p≤0.02 versus sham-Veh and sham-PTHrP groups by ANOVA.

In response to a glucose challenge at day 25 (Fig 3A), the PPx-veh mice were significantly glucose intolerant relative to both sham-operated groups, as assessed by area under the curve (AUC) (Fig 3A and 3B), confirming previous observations that PPx causes glucose intolerance [26]. However, the PPx-PTHrP mice were not glucose intolerant relative to either of the sham controls at day 25 (Fig 3A and 3B). By day 79, there was no significant difference in the glucose tolerance among the four groups of mice, as measured by AUC (Fig 3C and 3D). The effect of PTHrP(1–36) treatment on insulin sensitivity was examined at early (day 16) (Fig 3E and 3F) and late (day 74) (Fig 3G and 3H) time points. There was no significant change in insulin sensitivity among the four groups at either time point.

### PTHrP(1–36) enhances beta cell proliferation in PPx mice

**Fig 4:**
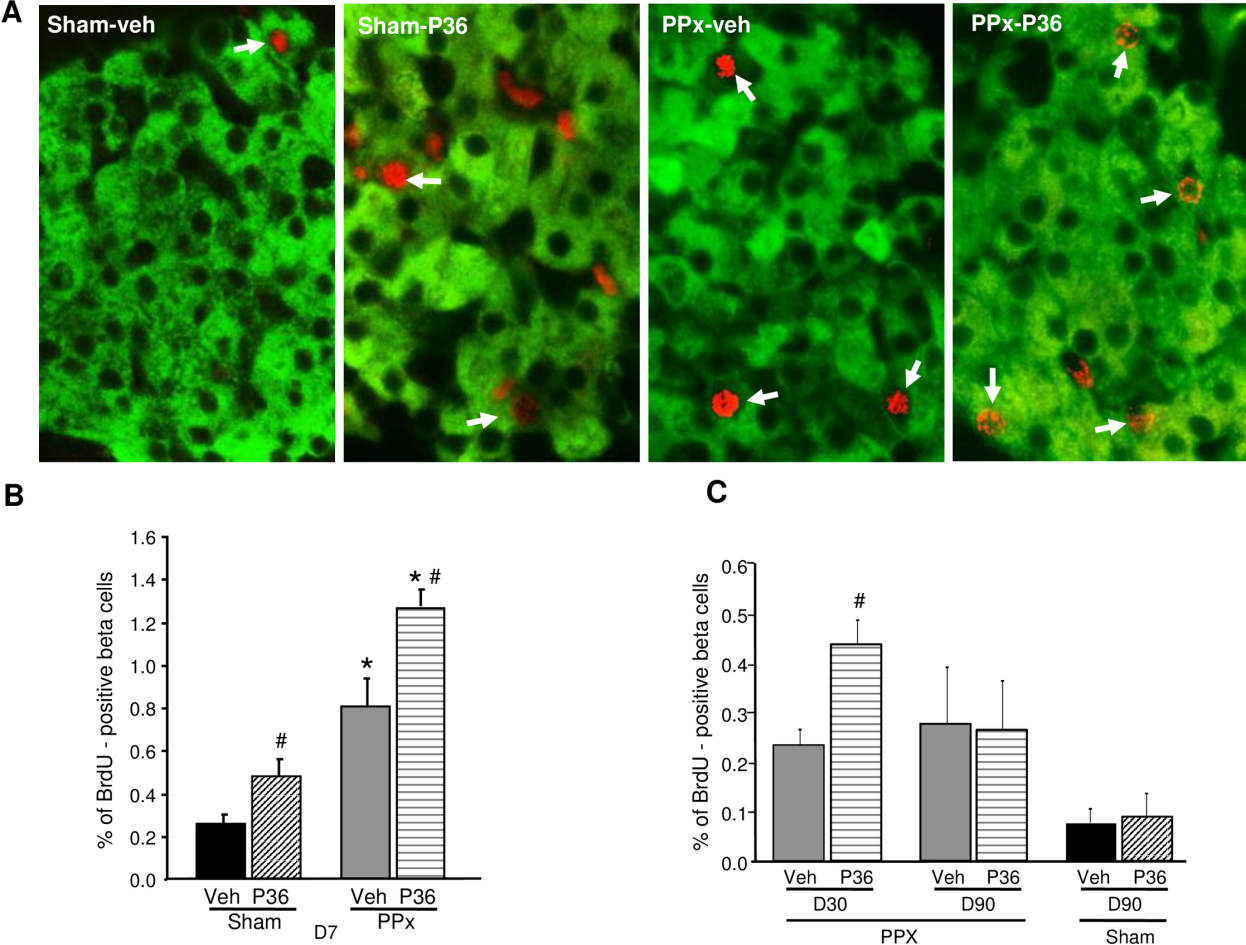
PTHrP(1–36) further increases beta cell proliferation induced by PPx. **(A)** Pancreatic sections from sham or PPx mice treated for 7 days with vehicle (veh) or PTHrP(1–36) (P36) and stained for insulin (green) and BrdU (red), with BrdU-positive beta cells marked by arrows. Quantification of the percentage of BrdU-positive beta cells after **(B)** 7 days (n = 5 mice/group), **(C)** 30 days (n = 4 mice/group) and 90 days (n = 6 mice/group) of treatment in the four groups of mice, as represented in Fig 1. Average of 1032±61 beta cells/pancreas was counted. Significance of * p<0.01 versus corresponding sham groups, and # p<0.05 versus corresponding Veh-treated groups, by ANOVA.

The observation that PTHrP increases beta cell proliferation *in vivo* under basal conditions [24], and PPx induces PTH1R expression (Fig 1D and 1E) led us to hypothesize that PTHrP(1–36) might further enhance beta cell regeneration after PPx. The effect of PTHrP(1–36) on beta cell proliferation was evaluated by quantifying the ratio of the BrdU (red) and insulin (green) double-positive cells to insulin-positive cells (Fig 4A). As observed previously under basal conditions [24], we found a significant 88% increase in the number of BrdU-positive beta cells in the sham-PTHrP compared to the sham-veh group at day 7 (0.48±0.17 versus 0.26±0.11, respectively) (Fig 4B). As expected [25], PPx by itself induced a striking three-fold increase in beta cell proliferation compared to sham-veh mice (0.81±0.28 versus 0.26±0.11, respectively). Most importantly, PTHrP(1–36) treatment significantly enhanced beta cell proliferation further by 57% in PPx-PTHrP versus PPx-veh mice at day 7 (1.28±0.19 versus 0.81±0.28, respectively) (Fig 4A and 4B). PTHrP(1–36) also increased beta cell proliferation in PPx mice, after 30 days of treatment (0.44±0.09 versus 0.21±0.1, PPx-PTHrP versus PPx-veh mice, respectively) (Fig 4C). However, by 90 days of treatment, PTHrP caused no additional increase in beta cell proliferation beyond PPx alone (Fig 4C).

**Fig 5:**
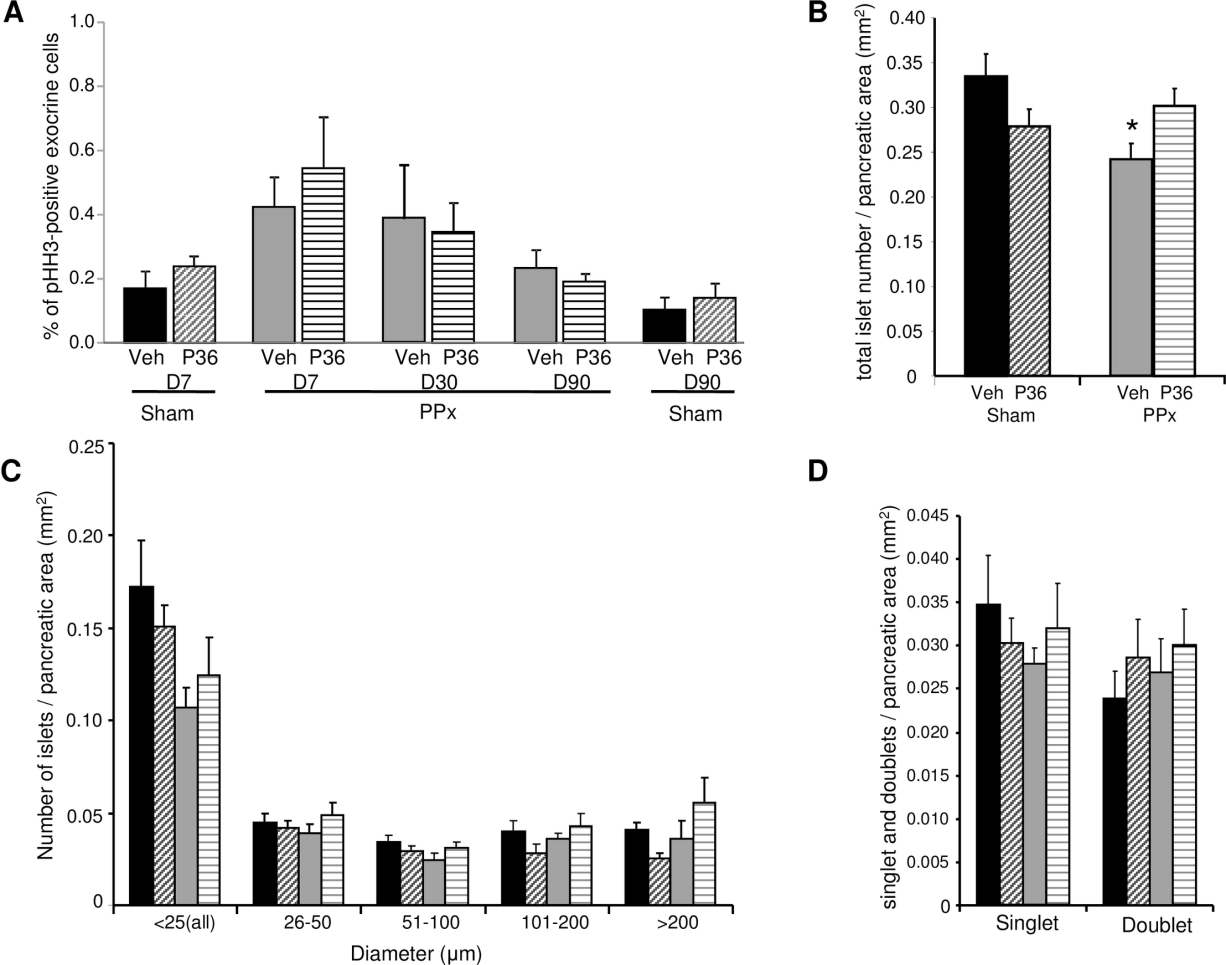
Exocrine cell proliferation, islet number and size distribution. **(A)** Quantification of the percentage of pHH3-positive exocrine cells in sham and PPx mice treated with vehicle (Veh) or PTHrP(1–36) (P36) for 7 days (D7, n = 5 mice/group), 30 days (D30, n = 4 mice/group) and 90 days (D90, n = 6 mice/group), as represented in Fig 1. Average of 3921±133 exocrine cells/pancreas was counted. **(B)** Total islet number/pancreatic area, **(C)** islet size distribution, counting average number of islets in size ranges from <25 to >200μm diameter/ pancreatic area, and **(D)** average number of singlet and doublet beta cell-containing islets/ pancreatic area, in the four groups of mice treated for 90 days, as represented in Fig 1 (n = 6 mice/group). Significance of * p<0.05 versus sham-veh group by ANOVA.

To assess whether the proliferative effect of PTHrP(1–36) was specific to beta cells, we measured exocrine cell proliferation in the pancreata of the four groups of mice at 7, 30 and 90 days. Although there was a trend towards an increase in exocrine cell proliferation with PPx in the PPx-Veh and PPx-PTHrP mice relative to the sham controls at day 7, the increase was not statistically significant (Fig 5A). Overall, neither PPx nor PTHrP treatment caused a significant change in exocrine cell replication at any of the three time points (Fig 5A, and data not shown).

### PTHrP(1–36) treatment increases beta cell mass in PPx mice

A detailed histomorphometric analysis of the pancreata, including total number and size distribution of islets, and beta cell mass, in the four groups of mice was undertaken at day 90. The total number of islets as a function of pancreatic area was similar in the sham-veh (0.34±0.03), sham-PTHrP (0.28±0.02), and PPx-PTHrP (0.30±0.02) mice. However, the total number of islets was significantly lower in the PPx-veh (0.24±0.02) mice compared to the sham-veh controls (Fig 5B). Analysis of the islet size distribution revealed no significant changes in islet size among the four groups of mice, although there was a trend towards an increase in the number of islets in the PPx-PTHrP versus PPx-veh mice in all size ranges (Fig 5C). The number of small islets (<25μm) including beta cell singlets or doublets, used as an indicator of islet neogenesis, was not significantly different among the four groups after 90 days of treatment (Fig 5D).

**Fig 6:**
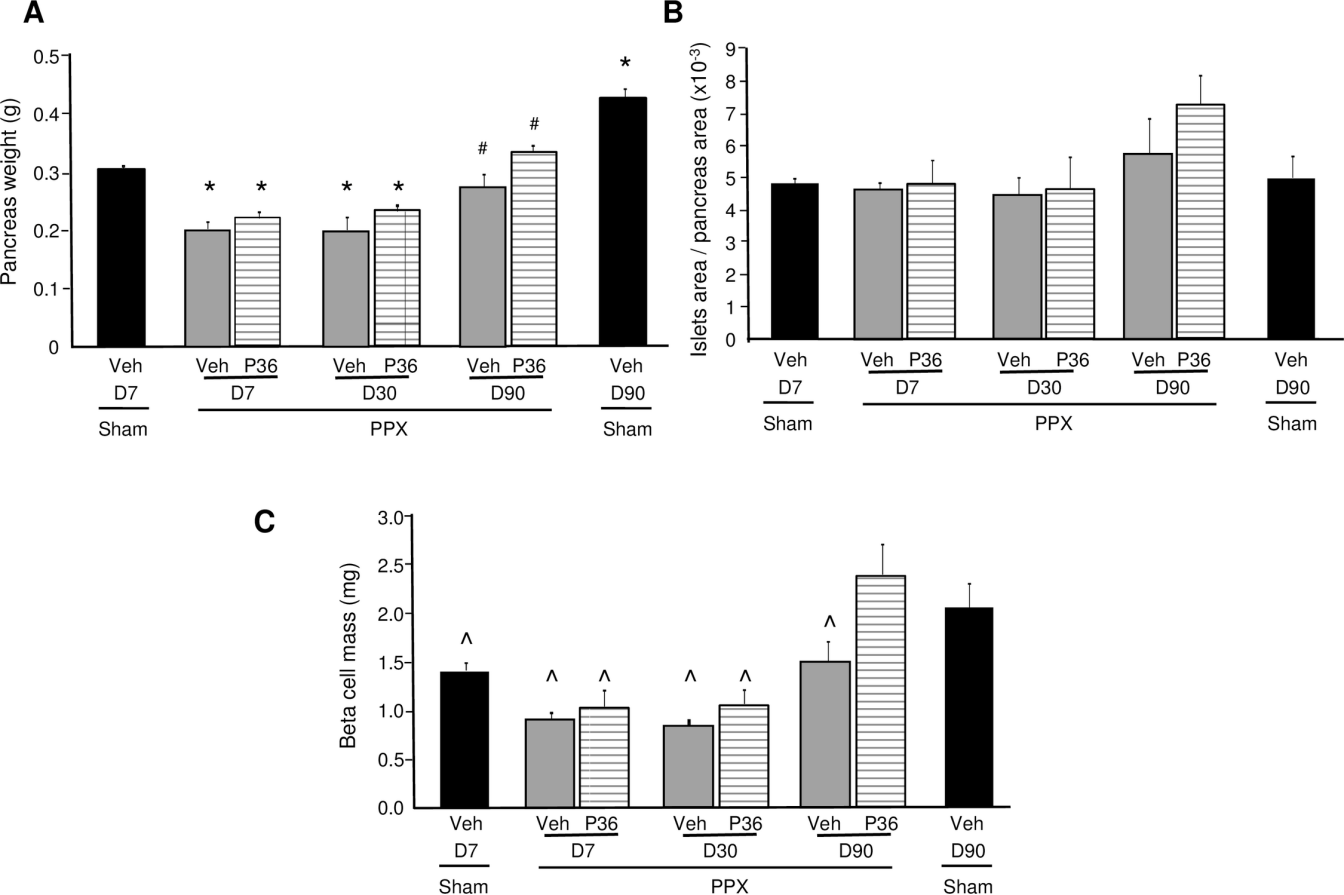
PTHrP(1–36) significantly enhances beta cell mass at 90 days in PPx mice. Histomorphometric analysis of pancreata in sham-veh (black bars) mice treated for 7 (D7) and 90 days (D90), and PPx-veh (grey bars) and PPx-PTHrP (P36) (horizontally-stippled bars) mice treated for 7 (D7, n = 5 mice/group), 30 (D30, n = 4 mice/group), and 90 (D90, n = 6 mice/group) days, measuring **(A)** pancreatic weight, **(B)** ratio of beta cell area/pancreatic area, and **(C)** beta cell mass. Significance of * p<0.05 versus sham-veh mice at D7, # p<0.05 versus sham-veh mice at D90, and **^** p≤0.05 versus PPx-PTHrP mice at D90, by ANOVA.

To examine whether the increase in beta cell proliferation observed in PTHrP(1–36)-treated PPx mice resulted in enhanced beta cell regeneration, beta cell mass was measured at 7, 30, and 90 days in these mice (Fig 6). As beta cell mass is a composite of pancreatic weight and the ratio of beta cell area/pancreatic area, these parameters were individually compared among the groups of mice. PTHrP(1–36) treatment did not significantly affect pancreatic weight in either the sham (data not shown), or the PPx mice, at 7, 30, or 90 days, although there was a trend towards an increase at 90 days in the PPx mice (Fig 6A). As expected, the remnant pancreas weight in both groups of PPx mice at days 7 and 30 was significantly lower than the sham-veh mice at day 7 (Fig 6A). By 90 days, the pancreas weight of the PPx-veh and PPx-PTHrP mice although similar to the weight of the sham-veh mice at day 7, was still significantly less than the sham-veh mice at day 90 (Fig 6A). The ratio of beta cell area/pancreatic area was not statistically different among the various groups (Fig 6B). However, this ratio tended to increase, in the PPx-PTHrP (7.3±0.9), compared to the PPx-veh (5.8±1.1) and sham-veh (5.0±0.7) mice at day 90 (Fig 6B).

The beta cell mass in the PPx groups at day 7, (PPx-veh 0.92±0.04mg and PPx-PTHrP 1.04±0.16mg) was decreased by approximately 30% relative to the sham-veh (1.41±0.07mg) mice (Fig 6C), as expected. Beta cell mass in the sham-PTHrP (1.47±0.16mg) mice was not different from sham-veh mice at day 7. At day 30, beta cell mass between the PPx-veh and PPx-PTHrP groups was not significantly different (Fig 6C). However, by day 90, beta cell mass in the PPx-PTHrP (2.4±0.31mg) mice was increased significantly relative to the PPx-veh (1.4±0.2mg) mice (Fig 6C), and had fully normalized as compared to the sham-veh (2.1±0.24mg) and sham-PTHrP (1.9±0.27mg) mice at day 90.

## Discussion

The current study demonstrates that PTHrP(1–36) potentiates beta cell regeneration, and accelerates the increase of beta cell mass through enhanced proliferation of beta cells. We used the 40% PPx model of beta cell regeneration since there is loss of both exocrine and endocrine tissues, without the accompanying deleterious effects of hyperglycemia or obvious inflammation [25]. Balb/c mice were used because their beta cell regenerative capacity is slower than other strains of mice [26]. The 160μg/kg PTHrP(1–36) dose regimen was based on our previously published data in normal mice [24], where we have shown a dose-dependent effect of PTHrP(1–36) with a maximal effect on beta cell proliferation at the dose used in the current study. We have previously demonstrated that PTHrP(1–36) enhances beta cell proliferation at 7 and 25 days of treatment. This was associated with an increase in beta cell mass by 25 days, without negatively impacting glucose homeostasis in Balb/c mice under basal conditions [24].

In this study, PTH1R levels were significantly increased in the islets of PPx mice, implying that exogenous PTHrP(1–36) has the potential to further enhance beta cell regeneration and/or glucose homeostasis after PPx. PTHrP did not significantly affect body weight, blood glucose, plasma insulin, or insulin sensitivity at early or later stages of treatment, in either the sham-operated or PPx mice, independently corroborating our previous findings in normal mice [24]. Although PPx-veh mice, as shown previously [26], were significantly glucose intolerant compared to either of the sham-control groups at day 25, the PPx-PTHrP mice were not, suggesting that PTHrP-treatment improves glucose tolerance at early stages of treatment under PPx conditions. By 79 days, there was no significant difference in glucose tolerance among the four groups. This is parallel to our previous observations in normal mice [24] where initial treatment with PTHrP improved glucose tolerance by seven days, but the improvement in glucose tolerance was lost after 25 days of PTHrP-treatment.

Both PTHrP(1–36) and PPx *per se* induced beta cell proliferation relative to sham-veh mice as early as seven days of treatment. Importantly, PTHrP-treatment further increased beta cell proliferation in PPx mice, at days 7 and 30, relative to PPx-veh mice. However, by 90 days, PTHrP-treatment did not result in a further increase in beta cell proliferation in PPx- or sham-operated mice. This could be due to down-regulation or desensitization of the PTH1R signaling in beta cells with long-term PTHrP treatment [28–29]. It is also possible that lower and/or more intermittent doses of PTHrP(1–36) may induce beta cell proliferation and/or improved glucose tolerance on a more sustained basis, as observed with the anabolic effect of PTHrP on bone [30–31]. Unlike its effect on the beta cell, PTHrP(1–36) did not increase exocrine cell proliferation in sham or PPx mice at 7, 30, or 90 days of treatment.

Islet histomorphometric analysis indicated a significantly lower number of total islets/pancreatic area at day 90 in the in the PPx-veh mice, but not in the PPx-PTHrP mice, relative to the sham-veh mice, suggesting enhanced regeneration of beta cells in the PPx-PTHrP group versus the PPx-veh group. PTHrP-treatment for 90 days did not cause a significant change in the number of small islets, which have been suggested to represent newly formed islets [32]. This may imply that PTHrP does not affect neogenesis, as expected in this PPx model in which proliferation of pre-existing beta cells has been shown to be the primary source of beta cell regeneration [9]. It is possible that PTHrP may increase the number of small islets in the early part of the treatment, and by day 90 the difference is no longer obvious. However, this seems unlikely, based on our finding in normal mice where PTHrP(1–36) did not affect number of small islets after 25 days of treatment [24]. Importantly, the increase in beta cell proliferation induced by PTHrP(1–36) in the early phase of treatment translated to a significant increase in beta cell mass in the PPx-PTHrP mice compared to the PPx-veh mice by day 90. This indicates that PTHrP(1–36) is indeed a beta cell regenerative factor.

PTHrP is a regenerative factor for other tissues and cell types, including in the regenerating deer antler, following peripheral nerve injury, and in bone and cartilage regeneration [33–36]. The current study adds the pancreatic beta cell as an additional target for PTHrP-induced regeneration. In this regard, although transcription factors and cell cycle molecules induce beta cell regeneration [37–38], there are few examples of exogenous peptides, such as glucagon-like peptide-1, and more recently, hepatocyte growth factor, that stimulate beta cell regeneration through proliferation of pre-existing beta cells [25–26].

Importantly, PTHrP and its receptor, PTH1R, are expressed in human islets and in human beta cells [13–15, 39]. Full-length PTHrP(1–139), as well as amino-terminal PTHrP(1–36), can enhance human beta cell proliferation and glucose-stimulated insulin secretion in human islets *in vitro* [39]. Based on the regenerative effect of PTHrP in PPx mice in the current study, our previous findings on the proliferative effect of PTHrP in normal mice under basal conditions [24], and importantly, the increase in human beta cell function and replication induced by PTHrP(1–36) in culture [39], we believe PTHrP(1–36) is a promising peptide for inducing beta cell proliferation and regeneration. The use of PTHrP(1–36) in clinical trials for the treatment of osteoporosis further attests to the safe use of this peptide in humans [27, 40]. Based on the established long-term anabolic effects of PTHrP on the skeleton [30–31], and now the beta cell, it is important to determine the proper regimen (dose, duration) of PTHrP(1–36) treatment on human beta cell regeneration. It is equally critical to understand its mechanism of action in the human beta cell, for optimal outcomes on beta cell growth, survival, and glucose homeostasis in the future. The proliferative, regenerative, pro-survival, and enhanced functional effects of PTHrP(1–36) on the beta cell may provide a useful therapeutic strategy to increase functional beta cell mass in the setting of diabetes in the future.

## References

[1] Boggi U, Rosati CM, Marchetti P (2013) Follow-up of secondary diabetic complications after pancreas transplantation. Curr Opin Organ Transplant 18:102–110 10.1097/MOT.0b013e32835c28c5 23283247

[2] Gremizzi C, Vergani A, Paloschi V, Secchi A (2010) Impact of pancreas transplantation on type 1 diabetes-related complications. Curr Opin Organ Transplant 15:119–123 10.1097/MOT.0b013e32833552bc 20010104

[3] McCall M, Shapiro AM (2012) Update on islet transplantation. Cold Spring Harb Perspect Med 2:a007823 10.1101/cshperspect.a007823 22762022 PMC3385934

[4] Teta M, Long SY, Wartschow LM, Rnakin MM, Kushner JA (2005) Very slow turnover of beta cells in aged adult mice. Diabetes 54:2557–2567 16123343 10.2337/diabetes.54.9.2557

[5] Tschen SI, Dhawan S, Gurlo T, Bhushan A (2009) Age-dependent decline in beta-cell proliferation restricts the capacity of beta-cell regeneration in mice. Diabetes 58:1312–1320 10.2337/db08-1651 19228811 PMC2682690

[6] Perl S, Kushner JA, Buchholz BA, Meeker AK, Stein GM, Hsieh M, et al (2010) Significant human beta-cell turnover is limited to the first three decades of life as determined by in vivo thymidine analog incorporation and radiocarbon dating. J Clin Endocrinol Metab 95:E234–E239 10.1210/jc.2010-0932 20660050 PMC3050099

[7] Rieck S, Kaestner KH (2010) Expansion of beta-cell mass in response to pregnancy. Trends Endocrinol Metab 21:151–158 10.1016/j.tem.2009.11.001 20015659 PMC3627215

[8] Sachdeva MM, Stoffers DA (2009) Minireview: Meeting the demand for insulin: molecular mechanisms of adaptive postnatal beta-cell mass expansion. Mol Endocrinol 23:747–758 10.1210/me.2008-0400 19196831 PMC2691682

[9] Dor Y, Brown J, Martinez OI, Melton DA (2004) Adult pancreatic beta-cells are formed by self-duplication rather than stem-cell differentiation. Nature 429:41–46 15129273 10.1038/nature02520

[10] Philbrick WM, Wysolmerski JJ, Galbraith S, Holt E, Orloff JJ, Yang KH, et al (1996) Defining the roles of parathyroid hormone-related protein in normal physiology. Physiol Rev 76:127–173 8592727 10.1152/physrev.1996.76.1.127

[11] Wysolmerski JJ (2012) Parathyroid hormone-related protein: an update. J Clin Endocrinol Metab 97:2947–2956 10.1210/jc.2012-2142 22745236 PMC3431578

[12] Fujinaka Y, Sipula D, Garcia-Ocaña A, Vasavada RC (2004) Characterization of mice doubly transgenic for parathyroid hormone-related protein and murine placental lactogen: a novel role for placental lactogen in pancreatic beta cell survival. Diabetes 53:3120–3130 15561942 10.2337/diabetes.53.12.3120

[13] Drucker DJ, Asa SL, Henderson J, Goltzman D (1989) The parathyroid hormone-like peptide gene is expressed in the normal and neoplastic human endocrine pancreas. Mol Endocrinol 3:1589–1595 2691879 10.1210/mend-3-10-1589

[14] Asa SL, Henderson J, Goltzman D, Drucker DJ (1990) Parathyroid hormone-like peptide in normal and neoplastic human endocrine tissues. J Clin Endocrinol Metab 71:1112–1118 2229275 10.1210/jcem-71-5-1112

[15] Sawada Y, Kameya T, Aizama T, Izumi T, Takeuchi T (2000) Proprotein-processing endoprotease furin and its substrate parathyroid hormone-related protein are coexpressed in insulinoma cells. Endocr Pathol 11:31–39 12114655 10.1385/ep:11:1:31

[16] Vasavada RC, Cavaliere C, D'Ercole AJ, Dann P, Burtis WJ, Madlener AL, et al (1996) Overexpression of parathyroid hormone-related protein in the pancreatic islets of transgenic mice causes islet hyperplasia, hyperinsulinemia, and hypoglycemia. J Biol Chem 271:1200–1208 8557651 10.1074/jbc.271.2.1200

[17] Porter SE, Sorenson RL, Dann P, Garcia-Ocana A, Stewart AF, Vasavada RC (1998) Progressive pancreatic islet hyperplasia in the islet-targeted, parathyroid hormone-related protein-overexpressing mouse. Endocrinology 139:3743–3751 9724026 10.1210/endo.139.9.6212

[18] Cebrian A, García-Ocaña A, Takane KK, Sipula D, Stewart AF, Vasavada RC (2002) Overexpression of parathyroid hormone-related protein inhibits pancreatic beta-cell death in vivo and in vitro. Diabetes 51:3003–3013 12351440 10.2337/diabetes.51.10.3003

[19] Vasavada RC, Wang L, Fujinaka Y, Takane KK, Rosa TC, Mellado-Gil JM, et al (2007) Protein kinase C-ζ markedly enhances beta cell proliferation: an essential role in growth factor-mediated beta cell mitogenesis. Diabetes 56:2732–2743 17686945 10.2337/db07-0461

[20] Plawner LL, Philbrick WM, Burtis WJ, Broadus AE, Stewart AF (1995) Cell type-specific secretion of parathyroid hormone-related protein via the regulated versus the constitutive secretory pathway. J Biol Chem 270:14078–14084 7775469 10.1074/jbc.270.23.14078

[21] Villanueva-Penacarrillo ML, Cancelas J, de Miguel F, Redondo A, Valín A, Valverde I, et al (1999) Parathyroid hormone-related peptide stimulates DNA synthesis and insulin secretion in pancreatic islets. J Endocrinol 163:403–408 10588813 10.1677/joe.0.1630403

[22] Sawada Y, Zhang B, Okajima F, Izumi T, Takeuchi T (2001) PTHrP increases pancreatic beta-cell-specific functions in well-differentiated cells. Mol Cell Endocrinol 182:265–275 11514060 10.1016/s0303-7207(01)00482-8

[23] Zhang B, Hosaka M, Sawada Y, Torii S, Mizutani S, Ogata M, et al (2003) Parathyroid hormone-related protein induces insulin expression through activation of MAP kinase-specific phosphatase-1 that dephosphorylates c-Jun NH2-terminal kinase in pancreatic beta-cells. Diabetes 52:2720–2730 14578290 10.2337/diabetes.52.11.2720

[24] Williams K, Abanquah D, Joshi-Gokhale S, Otero A, Lin H, Guthalu NK, et al (2011) Systemic and acute administration of parathyroid hormone-related peptide(1–36) stimulates endogenous beta cell proliferation while preserving function in adult mice. Diabetologia 54:2867–2877 10.1007/s00125-011-2260-z 21800111

[25] Alvarez-Perez JC, Ernst S, Demirci C, Casinelli GP, Mellado-Gil JM, Rausell-Palamos F, et al (2014) Hepatocyte Growth Factor/c-Met Signaling Is Required for ß-Cell Regeneration. Diabetes 63:216–223 10.2337/db13-0333 24089510 PMC3868042

[26] De León DD, Deng S, Madani R, Ahima RS, Drucker DJ, Stoffers DA (2003) Role of endogenous glucagon-like peptide-1 in islet regeneration after partial pancreatectomy. Diabetes 52:365–3671 12540609 10.2337/diabetes.52.2.365

[27] Horwitz MJ, Tedesco MB, Gundberg C, Garcia-Ocana A, Stewart AF (2003) Short-term, high-dose parathyroid hormone-related protein as a skeletal anabolic agent for the treatment of postmenopausal osteoporosis. J Clin Endocrinol Metab 88:569–575 12574182 10.1210/jc.2002-021122

[28] Guo J, Liu BY, Bringhurst FR (1997) Mechanisms of homologous and heterologous desensitization of PTH/PTHrP receptor signaling in LLC-PK1 cells. Am J Physiol 273:E383–E393 9277393 10.1152/ajpendo.1997.273.2.E383

[29] Vilardaga JP, Romero G, Friedman PA, Gardella TJ (2011) Molecular basis of parathyroid hormone receptor signaling and trafficking: a family B GPCR paradigm. Cell Mol Life Sci 68:1–13 10.1007/s00018-010-0465-9 20703892 PMC3568769

[30] Frolik CA, Black EC, Cain RL, Satterwhite JH, Brown-Augsburger PL, Sato M, et al (2003) Anabolic and catabolic bone effects of human parathyroid hormone (1–34) are predicted by duration of hormone exposure. Bone 33:372–379 13678779 10.1016/s8756-3282(03)00202-3

[31] Bellido T, Ali AA, Plotkin LI, Fu Q, Gubrij I, Roberson PK, et al (2003) Proteasomal degradation of Runx2 shortens parathyroid hormone-induced anti-apoptotic signaling in osteoblasts. A putative explanation for why intermittent administration is needed for bone anabolism. J Biol Chem 278:50259–50272 14523023 10.1074/jbc.M307444200

[32] Bonner-Weir S, Li WC, Ouziel-Yahalom L, Guo L, Weir GC, Sharma A (2010) Beta-cell growth and regeneration: replication is only part of the story. Diabetes 59:2340–2348 10.2337/db10-0084 20876724 PMC3279552

[33] Faucheux C, Nicholls BM, Allen S, Danks JA, Horton MA, Price JS (2004) Recapitulation of the parathyroid hormone-related peptide-Indian hedgehog pathway in the regenerating deer antler. Dev Dyn 231:88–97 15305289 10.1002/dvdy.20117

[34] Macica CM, Liang G, Lankford KL, Broadus AE (2006) Induction of parathyroid hormone-related peptide following peripheral nerve injury: role as a modulator of Schwann cell phenotype. Glia 53:637–648 16470617 10.1002/glia.20319

[35] de Castro LF, Lozano D, Dapía S, Portal-Núñez S, Caeiro JR, Gómez-Barrena E, et al (2010) Role of the N- and C-terminal fragments of parathyroid-hormone-related protein as putative therapies to improve bone regeneration under high glucocorticoid treatment. Tissue Eng 16:1157–1168 10.1089/ten.TEA.2009.0355 19860552

[36] Kudo S, Mizuta H, Takagi K, Hiraki Y (2011) Cartilaginous repair of full-thickness articular cartilage defects is induced by the intermittent activation of PTH/PTHrP signaling. Osteoarthritis Cartilage 19:886–894 10.1016/j.joca.2011.04.007 21571083

[37] Ackermann Misfeldt A, Costa RH, Gannon M (2008) Beta-cell proliferation, but not neogenesis, following 60% partial pancreatectomy is impaired in the absence of FoxM1. Diabetes 57:3069–3077 10.2337/db08-0878 18728229 PMC2570403

[38] Lee JH, Jo J, Hardikar AA, Periwal V, Rane SG (2010) Cdk4 regulates recruitment of quiescent beta-cells and ductal epithelial progenitors to reconstitute beta-cell mass. PLoS One 5:e8653 10.1371/journal.pone.0008653 20084282 PMC2801612

[39] Guthalu Kondegowda N, Joshi-Gokhale S, Harb G, Williams K, Zhang XY, Takane KK, et al (2010) Parathyroid hormone-related protein enhances human ß-cell proliferation and function with associated induction of cyclin-dependent kinase 2 and cyclin E expression. Diabetes 59:3131–3138 10.2337/db09-1796 20876711 PMC2992775

[40] Augustine M, Horwitz MJ (2013) Parathyroid Hormone and Parathyroid Hormone-Related Protein Analogs as Therapies for Osteoporosis. Curr Osteoporos Rep 11:400–406. 24078470 10.1007/s11914-013-0171-2 PMC3874264

